#  Influenza A(H5N6) Virus Reassortant, Southern China, 2014

**DOI:** 10.3201/eid2107.140838

**Published:** 2015-07

**Authors:** Hanqin Shen, Boliang Wu, Yimin Chen, Yingzuo Bi, Qingmei Xie

**Affiliations:** South China Agricultural University, Guangzhou, China (H. Shen, B. Wu, Y. Chen, Y. Bi, Q. Xie);; Key Laboratory of Animal Health Aquaculture and Environmental Control, Guangzhou (H. Shen, Q. Xie);; Ministry of Agriculture, Guangzhou (B. Wu, Q. Xie);; South China Collaborative Innovation Center for Poultry Disease Control and Product Safety, Guangzhou (Q. Xie)

**Keywords:** H5N6, avian influenza, phylogenetic analysis, viruses, influenza, China

**To the Editor:** Avian influenza A viruses generally do not cause disease in aquatic birds, the natural reservoir of these viruses (*1*). Influenza A(H5N6) was first isolated from mallards by García et al. in 1975 (*2*). Influenza viruses continue to evolve and reassort to generate novel, highly pathogenic viruses. Novel H5 highly pathogenic avian influenza virus subtypes, such as H5N2, H5N5, and H5N8, have been reported (*3*,*4*). Highly pathogenic influenza A viruses are endemic to many countries (http://www.oie.int/en/animal-health-in-the-world/update-on-avian-influenza/2015/), cause tremendous economic losses to the poultry industry, and represent a serious threat to public health.

In March 2014, an influenza A(H5N6) outbreak caused the death of 457 birds in Laos (http://www.oie.int/wahis_2/public%5C..%5Ctemp%5Creports/en_imm_0000015052_20140507_182757.pdf). During the same month, a flock of ducks in Guangdong Province in southern China exhibited typical respiratory signs of influenza A virus infection. This flock also had 70% decreased egg production and a slightly increased mortality rate. Throat swab specimens were taken from the symptomatic and dead ducks, and the samples were used to inoculate chicken embryos for virus isolation. Hemagglutination (HA) and neuraminidase (NA) inhibition assays were performed to identify the subtype of the isolated virus, which was designated A/duck/Guangdong/GD01/2014 (H5N6) (GD01/2014). The complete RNA genome was amplified by reverse transcription PCR and cloned into the pMD-19T vector for sequencing (*5*). The complete genome sequence of the GD01/2014 virus was submitted to GenBank (accession nos. KJ754142–KJ754149).

Multiple-sequence alignments showed that the HA gene of GD01/2014 shared 97.5% nt identity with A/wild duck/Shandong/628/2011 (H5N1) and NA genes shared 96.6% and 98.3% nt identity with A/swine/Guangdong/K6/2010 (H6N6) and A/duck/Shantou/1984/2007 (H6N6), respectively. All internal genes shared high levels of nucleotide identity (97.6%–98.5%) with A/wild duck/Fujian/2/2011(H5N1). The whole genes of GD01/2014 and the H5N6 viruses in Laos (LAO/2014) shared 98.2%–99.7% nt identity, indicating the same genotype. Phylogenetic analysis of the HA gene revealed that the isolated virus belonged to clade 2.3.4.6 (Technical Appendix Figure) (*6*). The NA gene of GD01/2014 was clustered with some H6N6 viruses circulating in China (Technical Appendix Figure, panel B). The 6 internal genes of GD01/2014 were closely related with A/wild duck/Fujian/2/2011(H5N1) and A/wild duck/Fujian/1/2011(H5N1) (Technical Appendix Figure, panels C–H). Phylogenetic analysis showed that all 8 genes of GD01/2014 and LAO/2014 were closely related although genetically distant from the earlier isolated H5N6 viruses (Technical Appendix Figure). These findings suggest that GD01/2014 and LAO/2014 are reassortants of wild duck H5N1 and H6N6 viruses, both of which have pathogenic and potential pandemic capacity in southern China. A previous report that H5N1 and H6N6 co-infected a duck suggests that GD01/2014 might be generated from the co-infection of H5N1 and H6N6 in the same host (*7*).

The intravenous pathogenicity index of GD01/2014 was 3.0, which indicates that the isolate is highly pathogenic for chickens. GD01/2014 had multiple basic amino acids (LRERRRKR/GLF) at the cleavage site between HA1 and HA2; this characteristic is typical of highly virulent influenza viruses (*8*). The HA protein contained E190, R220, G225, Q226, and G228 (H3 numbering) residues at the receptor-binding pockets, indicating that the virus preferentially binds to the sialic acid-2,3-NeuAcGal of the avian-like receptor (*9*). The HA protein has 7 potential N-glycosylation sites (PGSs); the HA1 protein has 5 PGSs; the HA2 protein has 2 PGSs. The NA protein of GD01/2014 and LAO/2014 had a deletion of 11 aa residues at positions 59–69 (N6 numbering) in the NA stalk region. Moreover, a deletion of 5 aa residues from positions 80–84 in the nonstructural 1 protein was found in GD01/2014 and LAO/2014. The position 627 and 701 of the polymerase basic 2 protein were E and D, respectively, characteristics of the avian influenza virus (*10*). 

In summary, in 2014, outbreaks of H5N6 virus occurred in China, Laos, and Vietnam and caused the deaths of infected humans in Sichuan province, China (http://www.oie.int/en/animal-health-in-the-world/update-on-avian-influenza/2014/; http://www.wpro.who.int/china/mediacentre/releases/2014/20140507/en/). We characterized the novel reassortant H5N6 virus in China and found that it was the same genome type as and was highly homologous with the H5N6 virus in Laos. The findings in this study are also supported by the previous genetic characterization of these viruses by Wong et al. (*11*). However, the adaptation, host range, and virulence of this reassortant H5N6 virus are still unclear and should be further investigated. Furthermore, the potential for infection, outbreaks, and pandemic in other poultry and mammals should be carefully monitored.

**Technical Appendix.** Phylogenetic relationship of genes of A/duck/Guangdong/GD01/2014(H5N6), a novel virus isolated from ducks in Guangdong Province in southern China during March 2014, compared with reference strains from GenBank.
