# Supplementary material for: Influenza A(H5N6) Virus Reassortant, Southern China, 2014
Source: Emerg Infect Dis. 2015 Jul;21(7):1261–2. doi: 10.3201/eid2107.140838 (PMC4480375; doi:10.3201/eid2107.140838)
Supplement: Supplementary file 1 — Technical Appendix. Phylogenetic relationship of genes of A/duck/Guangdong/GD01/2014(H5N6), a novel virus isolated from ducks in Guangdong Province in southern China during March 2014, compared with reference strains from GenBank. [file 14-0838-Techapp-s1.pdf]

Article DOI: http://dx.doi.org/10.3201/eid2107140838

Influenza A(H5N6) Virus Reassortant, Southern China, 2014

Technical Appendix

Technical Appendix Figure (following pages). Phylogenetic relationship of genes of A/duck/Guangdong/GD01/2014 (H5N6), a novel virus isolated from ducks in Guangdong Province in southern China during March 2014, compared with reference strains from GenBank. Phylogenetic relationship of specific genes are shown by panel: A) HA, hemagglutinin; B) NA, neuraminidase; C) PB2, polybasic protein 2; D) PB1, polybasic protein 1; E) PA, polymerase acidic protein; F) NP, nucleocapsid protein; G) M, matrix protein; H) NS, nonstructural protein. Triangles indicate the virus characterized in this study. Circles indicate other H5N6 viruses available in GenBank. The tree was constructed by using the neighbor-joining method of MEGA5.05 (www.megasoftware.net/) with 1,000 bootstrap replicates.

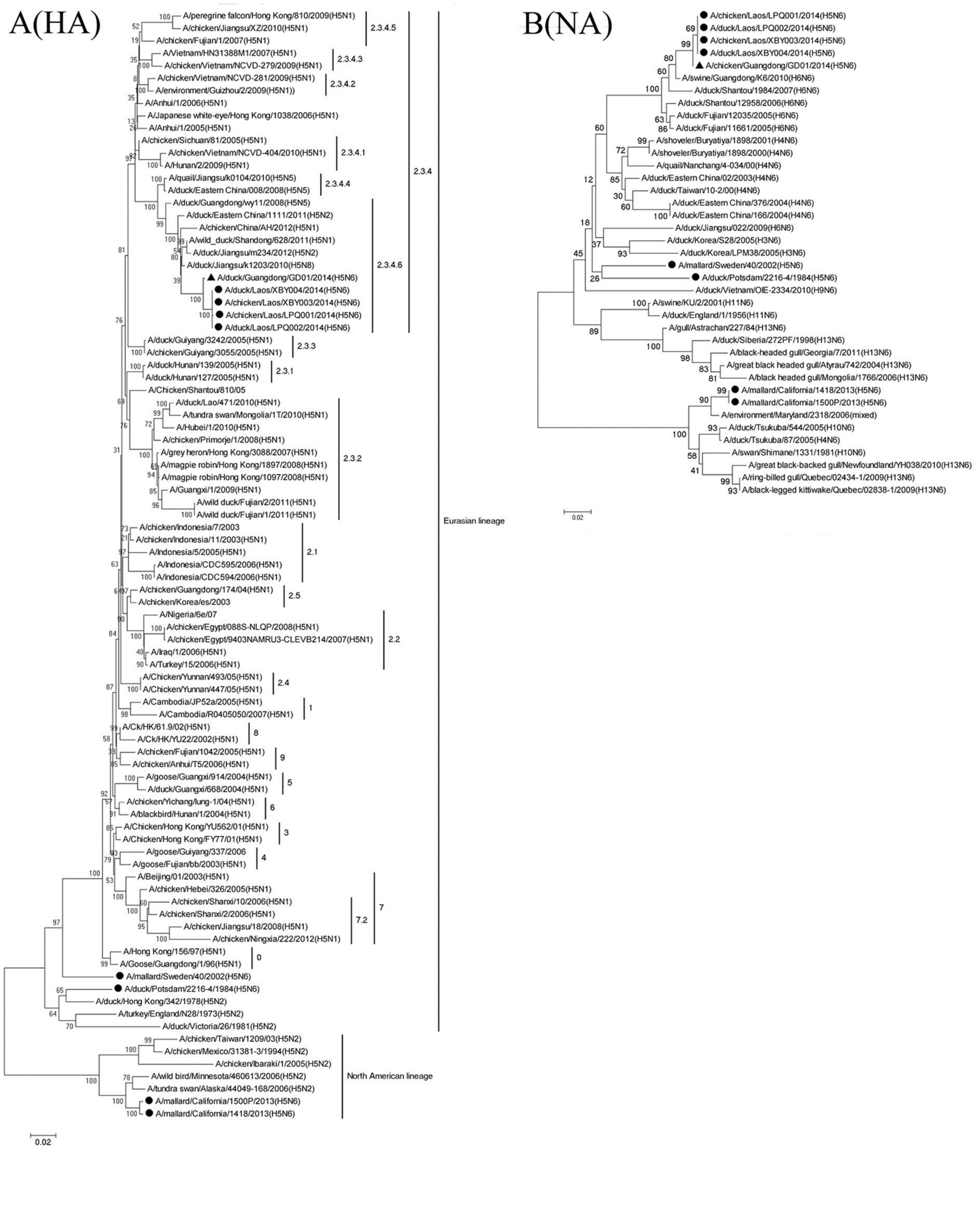
A(HA)
A/peregrine falcon/Hong Kong/810/2009(H5N1)
A/chicken/Jiangsu/XZ/2010(H5N1)
A/chicken/Fujian/1/2007(H5N1)
2.3.4.5
A/Vietnam/HN31388M1/2007(H5N1)
A/chicken/Vietnam/NCVD-279/2009(H5N1)
2.3.4.3
A/chicken/Vietnam/NCVD-281/2009(H5N1)
A/environment/Guizhou/2/2009(H5N1))
2.3.4.2
A/Anhui/1/2006(H5N1)
A/Japanese white-eye/Hong Kong/1038/2006(H5N1)
A/Anhui/1/2005(H5N1)
A/chicken/Sichuan/81/2005(H5N1)
A/chicken/Vietnam/NCVD-404/2010(H5N1)
A/Hunan/2/2009(H5N1)
2.3.4.1
A/quail/Jiangsu/k0104/2010(H5N5)
A/duck/Eastern China/008/2008(H5N5)
2.3.4.4
2.3.4
A/duck/Guangdong/wy11/2008(H5N5)
A/duck/Eastern China/1111/2011(H5N2)
A/chicken/China/AH/2012(H5N1)
A/wild_duck/Shandong/628/2011(H5N1)
A/duck/Jiangsu/m234/2012(H5N2)
A/duck/Jiangsu/k1203/2010(H5N8)
A/duck/Guangdong/GD01/2014(H5N6)
A/duck/Laos/XBY004/2014(H5N6)
A/chicken/Laos/XBY003/2014(H5N6)
A/chicken/Laos/LPQ001/2014(H5N6)
A/duck/Laos/LPQ002/2014(H5N6)
2.3.4.6
A/duck/Guiyang/3242/2005(H5N1)
A/chicken/Guiyang/3055/2005(H5N1)
2.3.3
A/duck/Hunan/139/2005(H5N1)
A/duck/Hunan/127/2005(H5N1)
2.3.1
A/Chicken/Shantou/810/05
A/duck/Lao/471/2010(H5N1)
A/tundra swan/Mongolia/1T/2010(H5N1)
A/Hubei/1/2010(H5N1)
A/chicken/Primorje/1/2008(H5N1)
A/grey heron/Hong Kong/3088/2007(H5N1)
A/magpie robin/Hong Kong/1897/2008(H5N1)
A/magpie robin/Hong Kong/1097/2008(H5N1)
A/Guangxi/1/2009(H5N1)
A/wild duck/Fujian/2/2011(H5N1)
A/wild duck/Fujian/1/2011(H5N1)
2.3.2
A/chicken/Indonesia/7/2003
A/chicken/Indonesia/11/2003(H5N1)
A/Indonesia/5/2005(H5N1)
A/Indonesia/CDC595/2006(H5N1)
A/Indonesia/CDC594/2006(H5N1)
2.1
A/chicken/Guangdong/174/04(H5N1)
A/chicken/Korea/es/2003
2.5
A/Nigeria/6e/07
A/chicken/Egypt/088S-NLQP/2008(H5N1)
A/chicken/Egypt/9403NAMRU3-CLEVB214/2007(H5N1)
A/Iraq/1/2006(H5N1)
A/Turkey/15/2006(H5N1)
2.2
A/Chicken/Yunnan/493/05(H5N1)
A/Chicken/Yunnan/447/05(H5N1)
2.4
A/Cambodia/JP52a/2005(H5N1)
A/Cambodia/R0405050/2007(H5N1)
1
A/Ck/HK/61.9/02(H5N1)
A/Ck/HK/YU22/2002(H5N1)
8
A/chicken/Fujian/1042/2005(H5N1)
A/chicken/Anhui/T5/2006(H5N1)
9
A/goose/Guangxi/914/2004(H5N1)
A/duck/Guangxi/668/2004(H5N1)
5
A/chicken/Yichang/lung-1/04(H5N1)
A/blackbird/Hunan/1/2004(H5N1)
6
A/Chicken/Hong Kong/YU562/01(H5N1)
A/Chicken/Hong Kong/FY77/01(H5N1)
3
A/goose/Guiyang/337/2006
A/goose/Fujian/bb/2003(H5N1)
4
A/Beijing/01/2003(H5N1)
A/chicken/Hebei/326/2005(H5N1)
A/chicken/Shanxi/10/2006(H5N1)
A/chicken/Shanxi/2/2006(H5N1)
A/chicken/Jiangsu/18/2008(H5N1)
A/chicken/Ningxia/222/2012(H5N1)
7.2
7
A/Hong Kong/156/97(H5N1)
A/Goose/Guangdong/1/96(H5N1)
0
A/mallard/Sweden/40/2002(H5N6)
A/duck/Potsdam/2216-4/1984(H5N6)
A/duck/Hong Kong/342/1978(H5N2)
A/turkey/England/N28/1973(H5N2)
A/duck/Victoria/26/1981(H5N2)
Eurasian lineage
A/chicken/Taiwan/1209/03(H5N2)
A/chicken/Mexico/31381-3/1994(H5N2)
A/chicken/Ibaraki/1/2005(H5N2)
A/wild bird/Minnesota/460613/2006(H5N2)
A/tundra swan/Alaska/44049-168/2006(H5N2)
A/mallard/California/1500P/2013(H5N6)
A/mallard/California/1418/2013(H5N6)
North American lineage
0.02
B(NA)
A/chicken/Laos/LPQ001/2014(H5N6)
A/duck/Laos/LPQ002/2014(H5N6)
A/chicken/Laos/XBY003/2014(H5N6)
A/duck/Laos/XBY004/2014(H5N6)
A/chicken/Guangdong/GD01/2014(H5N6)
A/swine/Guangdong/K6/2010(H6N6)
A/duck/Shantou/1984/2007(H6N6)
A/duck/Shantou/12958/2006(H6N6)
A/duck/Fujian/12035/2005(H6N6)
A/duck/Fujian/11661/2005(H6N6)
A/shoveler/Buryatiya/1898/2001(H4N6)
A/shoveler/Buryatiya/1898/2000(H4N6)
A/quail/Nanchang/4-034/00(H4N6)
A/duck/Eastern China/02/2003(H4N6)
A/duck/Taiwan/10-2/00(H4N6)
A/duck/Eastern China/376/2004(H4N6)
A/duck/Eastern China/166/2004(H4N6)
A/duck/Jiangsu/022/2009(H6N6)
A/duck/Korea/S28/2005(H3N6)
A/duck/Korea/LPM38/2005(H3N6)
A/mallard/Sweden/40/2002(H5N6)
A/duck/Potsdam/2216-4/1984(H5N6)
A/duck/Vietnam/OIE-2334/2010(H9N6)
A/swine/KU/2/2001(H11N6)
A/duck/England/1/1956(H11N6)
A/gull/Astrachan/227/84(H13N6)
A/duck/Siberia/272PF/1998(H13N6)
A/black-headed gull/Georgia/7/2011(H13N6)
A/great black headed gull/Atyrau/742/2004(H13N6)
A/black headed gull/Mongolia/1766/2006(H13N6)
A/mallard/California/1418/2013(H5N6)
A/mallard/California/1500P/2013(H5N6)
A/environment/Maryland/2318/2006(mixed)
A/duck/Tsukuba/544/2005(H10N6)
A/duck/Tsukuba/87/2005(H4N6)
A/swan/Shimane/1331/1981(H10N6)
A/great black-backed gull/Newfoundland/YH038/2010(H13N6)
A/ring-billed gull/Quebec/02434-1/2009(H13N6)
A/black-legged kittiwake/Quebec/02838-1/2009(H13N6)
0.02

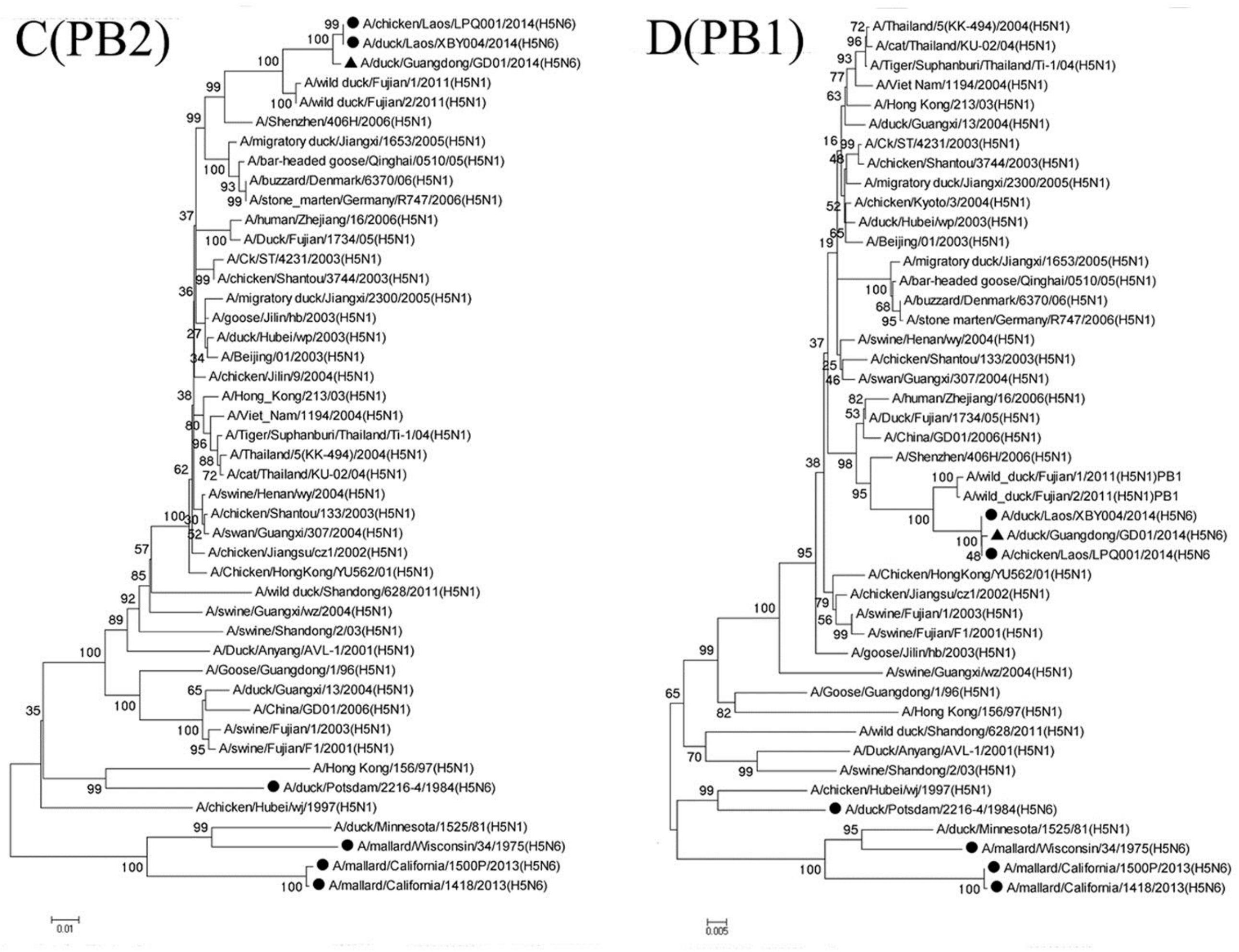

C(PB2)
A/chicken/Laos/LPQ001/2014(H5N6)
A/duck/Laos/XBY004/2014(H5N6)
A/duck/Guangdong/GD01/2014(H5N6)
A/wild duck/Fujian/1/2011(H5N1)
A/wild duck/Fujian/2/2011(H5N1)
A/Shenzhen/406H/2006(H5N1)
A/migratory duck/Jiangxi/1653/2005(H5N1)
A/bar-headed goose/Qinghai/0510/05(H5N1)
A/buzzard/Denmark/6370/06(H5N1)
A/stone_marten/Germany/R747/2006(H5N1)
A/human/Zhejiang/16/2006(H5N1)
A/Duck/Fujian/1734/05(H5N1)
A/Ck/ST/4231/2003(H5N1)
A/chicken/Shantou/3744/2003(H5N1)
A/migratory duck/Jiangxi/2300/2005(H5N1)
A/goose/Jilin/hb/2003(H5N1)
A/duck/Hubei/wp/2003(H5N1)
A/Beijing/01/2003(H5N1)
A/chicken/Jilin/9/2004(H5N1)
A/Hong_Kong/213/03(H5N1)
A/Viet_Nam/1194/2004(H5N1)
A/Tiger/Suphanburi/Thailand/Ti-1/04(H5N1)
A/Thailand/5(KK-494)/2004(H5N1)
A/cat/Thailand/KU-02/04(H5N1)
A/swine/Henan/wy/2004(H5N1)
A/chicken/Shantou/133/2003(H5N1)
A/swan/Guangxi/307/2004(H5N1)
A/chicken/Jiangsu/cz1/2002(H5N1)
A/Chicken/HongKong/YU562/01(H5N1)
A/wild duck/Shandong/628/2011(H5N1)
A/swine/Guangxi/wz/2004(H5N1)
A/swine/Shandong/2/03(H5N1)
A/Duck/Anyang/AVL-1/2001(H5N1)
A/Goose/Guangdong/1/96(H5N1)
A/duck/Guangxi/13/2004(H5N1)
A/China/GD01/2006(H5N1)
A/swine/Fujian/1/2003(H5N1)
A/swine/Fujian/F1/2001(H5N1)
A/Hong Kong/156/97(H5N1)
A/duck/Potsdam/2216-4/1984(H5N6)
A/chicken/Hubei/wj/1997(H5N1)
A/duck/Minnesota/1525/81(H5N1)
A/mallard/Wisconsin/34/1975(H5N6)
A/mallard/California/1500P/2013(H5N6)
A/mallard/California/1418/2013(H5N6)
0.01
D(PB1)
A/Thailand/5(KK-494)/2004(H5N1)
A/cat/Thailand/KU-02/04(H5N1)
A/Tiger/Suphanburi/Thailand/Ti-1/04(H5N1)
A/Viet Nam/1194/2004(H5N1)
A/Hong Kong/213/03(H5N1)
A/duck/Guangxi/13/2004(H5N1)
A/Ck/ST/4231/2003(H5N1)
A/chicken/Shantou/3744/2003(H5N1)
A/migratory duck/Jiangxi/2300/2005(H5N1)
A/chicken/Kyoto/3/2004(H5N1)
A/duck/Hubei/wp/2003(H5N1)
A/Beijing/01/2003(H5N1)
A/migratory duck/Jiangxi/1653/2005(H5N1)
A/bar-headed goose/Qinghai/0510/05(H5N1)
A/buzzard/Denmark/6370/06(H5N1)
A/stone marten/Germany/R747/2006(H5N1)
A/swine/Henan/wy/2004(H5N1)
A/chicken/Shantou/133/2003(H5N1)
A/swan/Guangxi/307/2004(H5N1)
A/human/Zhejiang/16/2006(H5N1)
A/Duck/Fujian/1734/05(H5N1)
A/China/GD01/2006(H5N1)
A/Shenzhen/406H/2006(H5N1)
A/wild_duck/Fujian/1/2011(H5N1)PB1
A/wild_duck/Fujian/2/2011(H5N1)PB1
A/duck/Laos/XBY004/2014(H5N6)
A/duck/Guangdong/GD01/2014(H5N6)
A/chicken/Laos/LPQ001/2014(H5N6
A/Chicken/HongKong/YU562/01(H5N1)
A/chicken/Jiangsu/cz1/2002(H5N1)
A/swine/Fujian/1/2003(H5N1)
A/swine/Fujian/F1/2001(H5N1)
A/goose/Jilin/hb/2003(H5N1)
A/swine/Guangxi/wz/2004(H5N1)
A/Goose/Guangdong/1/96(H5N1)
A/Hong Kong/156/97(H5N1)
A/wild duck/Shandong/628/2011(H5N1)
A/Duck/Anyang/AVL-1/2001(H5N1)
A/swine/Shandong/2/03(H5N1)
A/chicken/Hubei/wj/1997(H5N1)
A/duck/Potsdam/2216-4/1984(H5N6)
A/duck/Minnesota/1525/81(H5N1)
A/mallard/Wisconsin/34/1975(H5N6)
A/mallard/California/1500P/2013(H5N6)
A/mallard/California/1418/2013(H5N6)
0.005

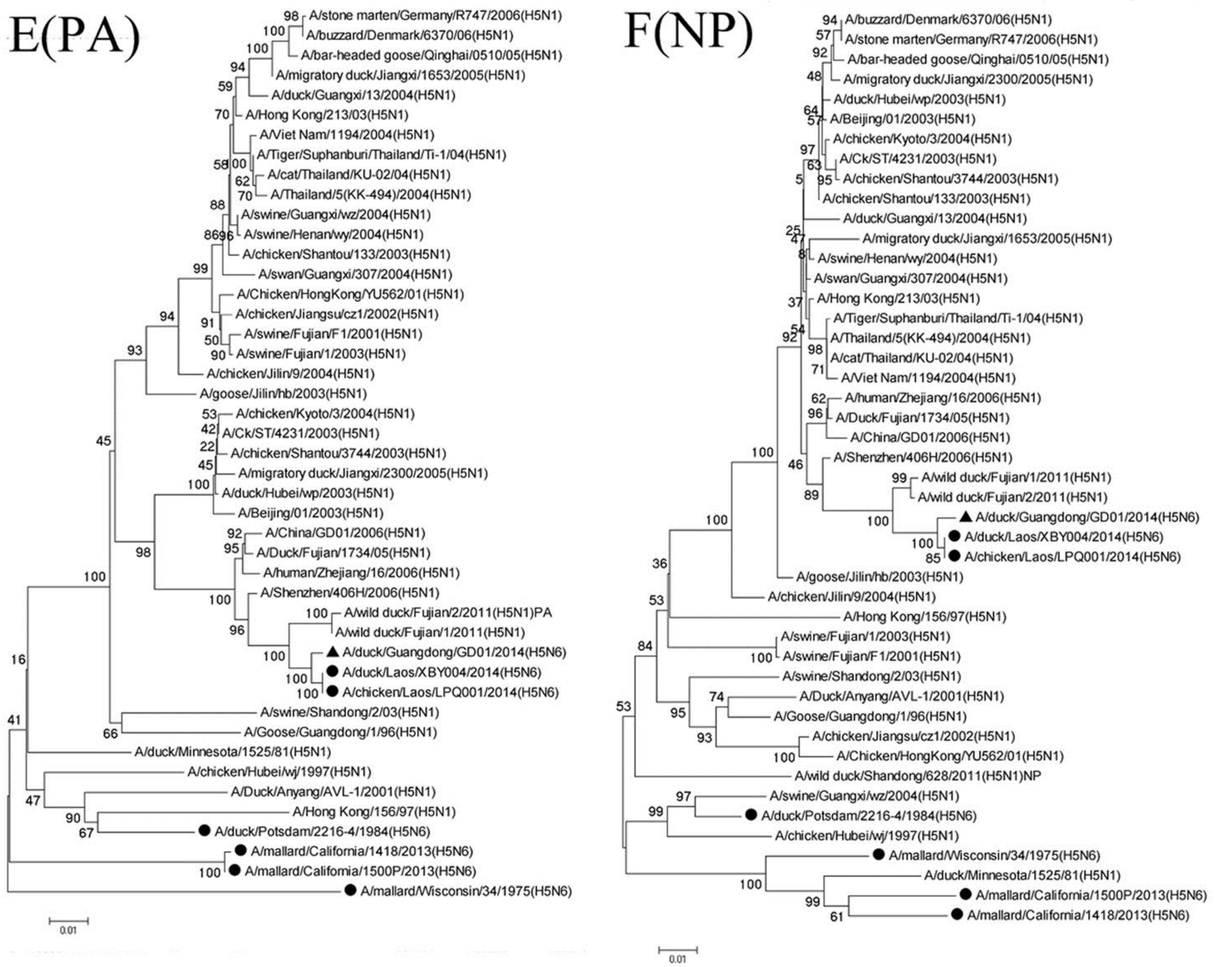
E(PA)
A/stone marten/Germany/R747/2006(H5N1)
A/buzzard/Denmark/6370/06(H5N1)
A/bar-headed goose/Qinghai/0510/05(H5N1)
A/migratory duck/Jiangxi/1653/2005(H5N1)
A/duck/Guangxi/13/2004(H5N1)
A/Hong Kong/213/03(H5N1)
A/Viet Nam/1194/2004(H5N1)
A/Tiger/Suphanburi/Thailand/Ti-1/04(H5N1)
A/cat/Thailand/KU-02/04(H5N1)
A/Thailand/5(KK-494)/2004(H5N1)
A/swine/Guangxi/wz/2004(H5N1)
A/swine/Henan/wy/2004(H5N1)
A/chicken/Shantou/133/2003(H5N1)
A/swan/Guangxi/307/2004(H5N1)
A/Chicken/HongKong/YU562/01(H5N1)
A/chicken/Jiangsu/cz1/2002(H5N1)
A/swine/Fujian/F1/2001(H5N1)
A/swine/Fujian/1/2003(H5N1)
A/chicken/Jilin/9/2004(H5N1)
A/goose/Jilin/hb/2003(H5N1)
A/chicken/Kyoto/3/2004(H5N1)
A/Ck/ST/4231/2003(H5N1)
A/chicken/Shantou/3744/2003(H5N1)
A/migratory duck/Jiangxi/2300/2005(H5N1)
A/duck/Hubei/wp/2003(H5N1)
A/Beijing/01/2003(H5N1)
A/China/GD01/2006(H5N1)
A/Duck/Fujian/1734/05(H5N1)
A/human/Zhejiang/16/2006(H5N1)
A/Shenzhen/406H/2006(H5N1)
A/wild duck/Fujian/2/2011(H5N1)PA
A/wild duck/Fujian/1/2011(H5N1)
A/duck/Guangdong/GD01/2014(H5N6)
A/duck/Laos/XBY004/2014(H5N6)
A/chicken/Laos/LPQ001/2014(H5N6)
A/swine/Shandong/2/03(H5N1)
A/Goose/Guangdong/1/96(H5N1)
A/duck/Minnesota/1525/81(H5N1)
A/chicken/Hubei/wj/1997(H5N1)
A/Duck/Anyang/AVL-1/2001(H5N1)
A/Hong Kong/156/97(H5N1)
A/duck/Potsdam/2216-4/1984(H5N6)
A/mallard/California/1418/2013(H5N6)
A/mallard/California/1500P/2013(H5N6)
A/mallard/Wisconsin/34/1975(H5N6)
0.01
F(NP)
A/buzzard/Denmark/6370/06(H5N1)
A/stone marten/Germany/R747/2006(H5N1)
A/bar-headed goose/Qinghai/0510/05(H5N1)
A/migratory duck/Jiangxi/2300/2005(H5N1)
A/duck/Hubei/wp/2003(H5N1)
A/Beijing/01/2003(H5N1)
A/chicken/Kyoto/3/2004(H5N1)
A/Ck/ST/4231/2003(H5N1)
A/chicken/Shantou/3744/2003(H5N1)
A/chicken/Shantou/133/2003(H5N1)
A/duck/Guangxi/13/2004(H5N1)
A/migratory duck/Jiangxi/1653/2005(H5N1)
A/swine/Henan/wy/2004(H5N1)
A/swan/Guangxi/307/2004(H5N1)
A/Hong Kong/213/03(H5N1)
A/Tiger/Suphanburi/Thailand/Ti-1/04(H5N1)
A/Thailand/5(KK-494)/2004(H5N1)
A/cat/Thailand/KU-02/04(H5N1)
A/Viet Nam/1194/2004(H5N1)
A/human/Zhejiang/16/2006(H5N1)
A/Duck/Fujian/1734/05(H5N1)
A/China/GD01/2006(H5N1)
A/Shenzhen/406H/2006(H5N1)
A/wild duck/Fujian/1/2011(H5N1)
A/wild duck/Fujian/2/2011(H5N1)
A/duck/Guangdong/GD01/2014(H5N6)
A/duck/Laos/XBY004/2014(H5N6)
A/chicken/Laos/LPQ001/2014(H5N6)
A/goose/Jilin/hb/2003(H5N1)
A/chicken/Jilin/9/2004(H5N1)
A/Hong Kong/156/97(H5N1)
A/swine/Fujian/1/2003(H5N1)
A/swine/Fujian/F1/2001(H5N1)
A/swine/Shandong/2/03(H5N1)
A/Duck/Anyang/AVL-1/2001(H5N1)
A/Goose/Guangdong/1/96(H5N1)
A/chicken/Jiangsu/cz1/2002(H5N1)
A/Chicken/HongKong/YU562/01(H5N1)
A/wild duck/Shandong/628/2011(H5N1)NP
A/swine/Guangxi/wz/2004(H5N1)
A/duck/Potsdam/2216-4/1984(H5N6)
A/chicken/Hubei/wj/1997(H5N1)
A/mallard/Wisconsin/34/1975(H5N6)
A/duck/Minnesota/1525/81(H5N1)
A/mallard/California/1500P/2013(H5N6)
A/mallard/California/1418/2013(H5N6)
0.01

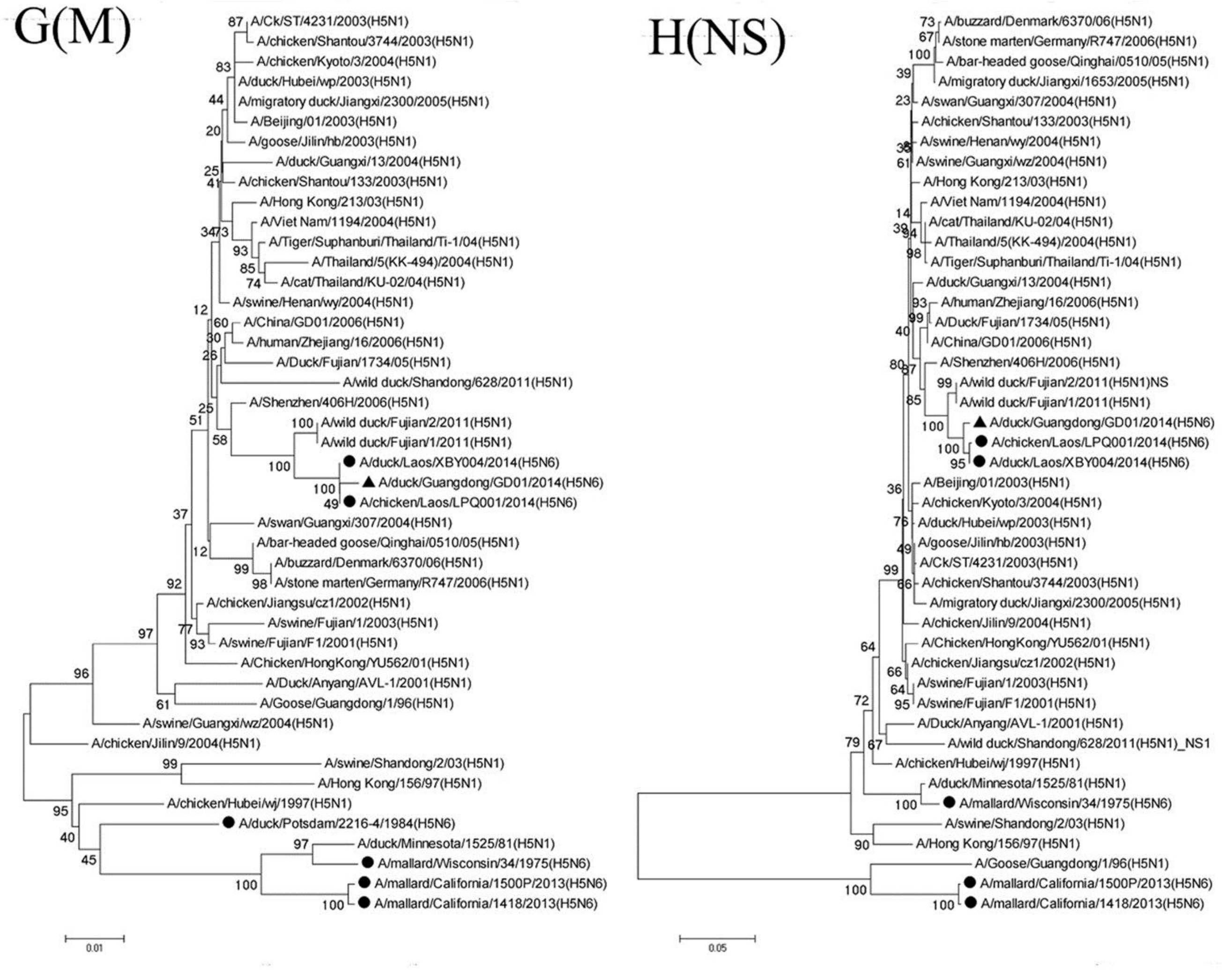
G(M)
H(NS)
0.01
0.05
